# Rapid and effective enrichment of mononuclear cells from blood using acoustophoresis

**DOI:** 10.1038/s41598-017-17200-9

**Published:** 2017-12-07

**Authors:** Anke Urbansky, Pelle Ohlsson, Andreas Lenshof, Fabio Garofalo, Stefan Scheding, Thomas Laurell

**Affiliations:** 1 Division of Nanobiotechnology, Department of Biomedical Engineering, Lund University, 221 00 Lund, Sweden; 2 AcouSort AB, Medicon Village, 223 81 Lund, Sweden; 3 Lund Stem Cell Center, Lund University, 221 00 Lund, Sweden; 4 Division of Molecular Hematology, Department of Laboratory Medicine, Lund University, 221 00 Lund, Sweden; 5 Department of Hematology, Skåne University Hospital, 222 41 Lund, Sweden; 6 Department of Biomedical Engineering, Dongguk University, 04620 Seoul, South Korea

## Abstract

Effective separation methods for fractionating blood components are needed for numerous diagnostic and research applications. This paper presents the use of acoustophoresis, an ultrasound based microfluidic separation technology, for label-free, gentle and continuous separation of mononuclear cells (MNCs) from diluted whole blood. Red blood cells (RBCs) and MNCs behave similar in an acoustic standing wave field, compromising acoustic separation of MNC from RBC in standard buffer systems. However, by optimizing the buffer conditions and thereby changing the acoustophoretic mobility of the cells, we were able to enrich MNCs relative to RBCs by a factor of 2,800 with MNC recoveries up to 88%. The acoustophoretic microchip can perform cell separation at a processing rate of more than 1 × 10^5^ cells/s, corresponding to 5 µl/min undiluted whole blood equivalent. Thus, acoustophoresis can be easily integrated with further down-stream applications such as flow cytometry, making it a superior alternative to existing MNC isolation techniques.

## Introduction

Mononuclear cells (MNCs), i.e. lymphocytes and monocytes, as part of the white blood cell population (WBC), play a critical role in the human immune system and are important in a variety of clinical and research applications. Working with MNCs often requires enrichment or isolation of the cells which can be challenging due to their low abundance of less than 0.06% of all blood cells^1^.

Separation of MNCs is commonly performed in batch processes either by density gradient centrifugation or red blood cell (RBC) lysis and centrifugation, both involving several manual handling steps. Alternatives to replace these labor-intensive methods are intensely researched within the microfluidics field. Miniaturized and automated fluid handling holds great promise of reducing several of the shortcomings encountered in macroscale handling of cell samples^2^. The deterministic behavior of fluids governed by the laminar flow conditions in microscale devices has driven the development of a wide range of modalities for separating cells, in which separation performances take advantages of physical scaling laws, but many times at the expense of system throughput^3–9^.

A key area of interest has been to develop methods that can differentiate cells solely based on their intrinsic parameters, which would enable a label free separation of the target cells and thereby reduce manual handling steps and costs. To achieve this goal, the microfluidic systems have to be designed to utilize differences in physical properties of the cells such as size, shape, density, electrical charge and deformability^2^. The benefits of these microfluidic concepts can then be fully realized by integration with downstream unit operations or other lab-on-a chip devices^5,10,11^.

**Table 1:** Label-free, continuous separation of WBC from blood using microfluidics.

Method		Dilution factor	WBC Separation efficiency	RBC depletion	Purity		WBC Enrichment	Sample throughput**
					WBC	MNC		
Cross-Flow filter	8	Undiluted	~98%	~99.975%	~70.5%	~28%*	~2000	0.06 µl/min
	12	Undiluted	~97.2%	NA	~96.9%	~39%*	NA	0.33 µl/min
Hydrodynamic filtration	13	10x	NA	NA	~3.6%	~1.4%*	~29	2 µl/min
Hydrophoretic filtration	15	20x (rat blood)	NA	NA	~58%	~23.2%*	~210	0.05 µl/min
Deterministic lateral displacement	9	Undiluted	~96% (WBC)~95% (MNC)	~99.1%	~9%	~5.5%	~110	0.018 µl/min
Microfiltration using rarchets	26	Undiluted	~98% (WBC)	~100%	~100%	~40%*	NA	0.083 µl/min
Inertial focusing	16	500x	~95%	~94%	NA	NA	NA	3.6 µl/min
	18	400x	~89.7%	~99.8%	~91%	~36.4%*	NA	0.375 µl/min
	19	20x	NA	NA	~48%	~19.2%*	NA	240 µl/min (30 µl/min per channel)
Dielectrophoresis	21	5x	~92.1%	~87%	NA	NA	NA	0.16 µl/min
Leukocyte margination	22	Undiluted	NA	NA	NA	NA	~34	NA
Continuous erythrocyte lysis	23	Undiluted	~100%	>99.5%	NA	NA	NA	0.5 µl/min
	24	10x	~99%	NA	NA	NA	NA	100 µl/min
Slanted hydrodynamic filtration	27	20x	~85%	NA	~80%	~32%*	NA	2 µl/min
Acoustophoresis	This work	20x	>43% (WBC) >87% (MNC)	>99.95%	~54%	~53%	~1000 (WBC) ~2800 (MNC)	5 µl/min

*Calculated based on assumption that ~40% of WBC are MNCs^25^

**Whole blood equivalent.

When addressing label-free and continuous WBC separation from blood using microfluidics, different working principles have been proposed including cross-flow filtration^8,12^, hydrodynamic filtration^13,14^, hydrophoretic filtration^15^, deterministic lateral displacement^9^, inertial focusing^16–19^, dielectrophoresis^20,21^, leukocyte margination^22^, and erythrocyte lysis^23,24^. However, most of the microfluidic devices reported thus far are either not sufficiently efficient in terms of separation performance or operate at low throughput rates ranging from 0.018 µl/min to 2 µl/min of undiluted blood^8,9,13^ (Table 1). Furthermore, to our understanding, none of the described methods allows for direct separation of MNCs from whole blood with acceptable purities of the MNC fraction.

Acoustophoresis, as an alternative microfluidic cell handling technique, offers a label-free and continuous cell separation that provides both high throughput and good separation performance for bioanalytical and medical applications^28–31^. Typically, an ultrasonic standing half wave is generated across a microchannel, in which acoustic radiation forces induce a movement of suspended cells or particles either towards the pressure node in the center of the channel or towards the pressure anti-node at the sidewalls. The magnitude and direction of the radiation force is dependent on the physical properties of the cells such as size, density and compressibility in relation to the surrounding medium^32^. In an aqueous system, denser particles, such as cells, are typically focused towards the pressure node while less dense particles, such as lipids, move to the pressure anti-node^33,34^. Particles with the same acoustic properties can be separated based on their size, as the acoustic radiation force scales with the particle volume and hence larger particles move faster than smaller particles^35^. Size based separation was successfully shown for a variety of clinical relevant applications such as separation of lymphocytes from granulocytes^36^, isolation of tumor cells^37^, separation of WBCs from platelets^38^, cell cycle phase synchronization in mammalian cells^39^ and isolation of bacteria in blood from sepsis patients^10^. Furthermore, acoustophoresis has been shown to be a gentle method that does not affect the viability and proliferative capacity of acoustically-separated cells^38,40–43^.

There is a clear unmet need for a rapid, simple and efficient method to separate MNC from blood, with at least a 3-log reduction of the RBC fraction and high MNC recovery above 80%, as an alternative to current labor-intensive density gradient centrifugation or RBC lysis and centrifugation^44^. Acoustophoresis based cell separation holds promise of addressing this shortcoming in view of the significant size differences between the two cell types. However, acoustophoretic WBC isolation experiments indicated that WBC and RBC displayed to a large extent overlapping acoustophoretic mobilities when diluted in the most commonly used buffer systems, such as phosphate buffered saline (PBS)^35^, which has so far prevented the use of acoustophoresis to generate pure MNC populations from blood for bioanalytical and diagnostic purposes.

In this paper, we report the development of an acoustophoresis protocol that enables the efficient and rapid separation of MNCs from diluted whole blood. The acoustophoresis chip was based on the earlier reported design by Augustsson^37^ with a 2-dimensional pre-alignment zone preceding the separation zone but was modified by increasing the channel length (both pre-focusing channel, from 10 mm to 26 mm and separation channel, from 20 mm to 43 mm) in order to enable an increased sample flow rate. Separation was realized by optimizing the acoustic properties of the suspending medium, thereby changing the acoustophoretic mobility of MNCs versus RBCs. This method has a much higher separation efficiency combined with higher throughput than previous reports on the label-free microfluidic separation of WBC components from blood^8,9,13,16,21^. The gentle, label-free and continuous acoustophoresis separation protocol developed herein presents an important step towards integration of downstream diagnostic applications such as direct analysis of MNCs in flow cytometry.

## Results and discussion

### Optimizing the acoustic properties of the suspending medium

**Figure 1:**
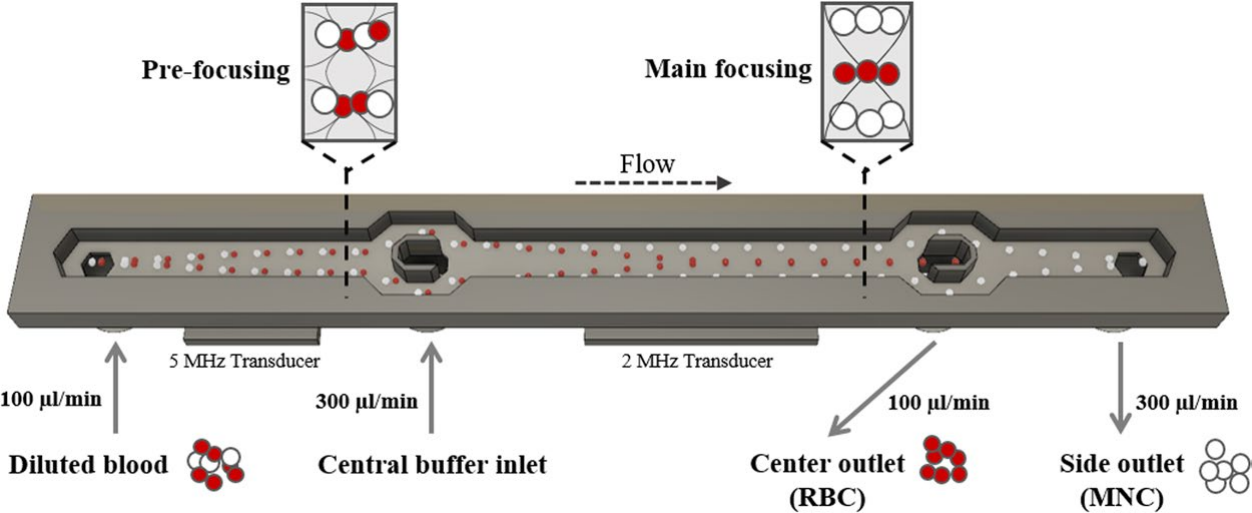
Microfluidic chip design. Diluted blood is injected into the pre-focusing channel where cells are pre-aligned using a 5 MHz transducer. The transducer generates an acoustic pressure node from top to bottom as well as a double node across the width of the channel, aligning the cells in height and width into two parallel bands. Pre-aligned blood is bifurcated at the v-shaped flow splitter around the central buffer inlet and enters the main focusing channel close to the channel wall. Due to the pre-alignment, cells are starting at identical positions and at the same flow speed, enhancing the resolution of the separation. In the main focusing channel, a 2 MHz transducer creates an acoustic standing wave field with a pressure node in the center of the channel, inducing movement of the cells depending on their acoustophysical properties. Cells with high acoustic mobility are moved faster to the channel center and can be collected in the center outlet while cells with low acoustic mobility stay close to the channel wall and are collected at the side outlet.

**Figure 2:**
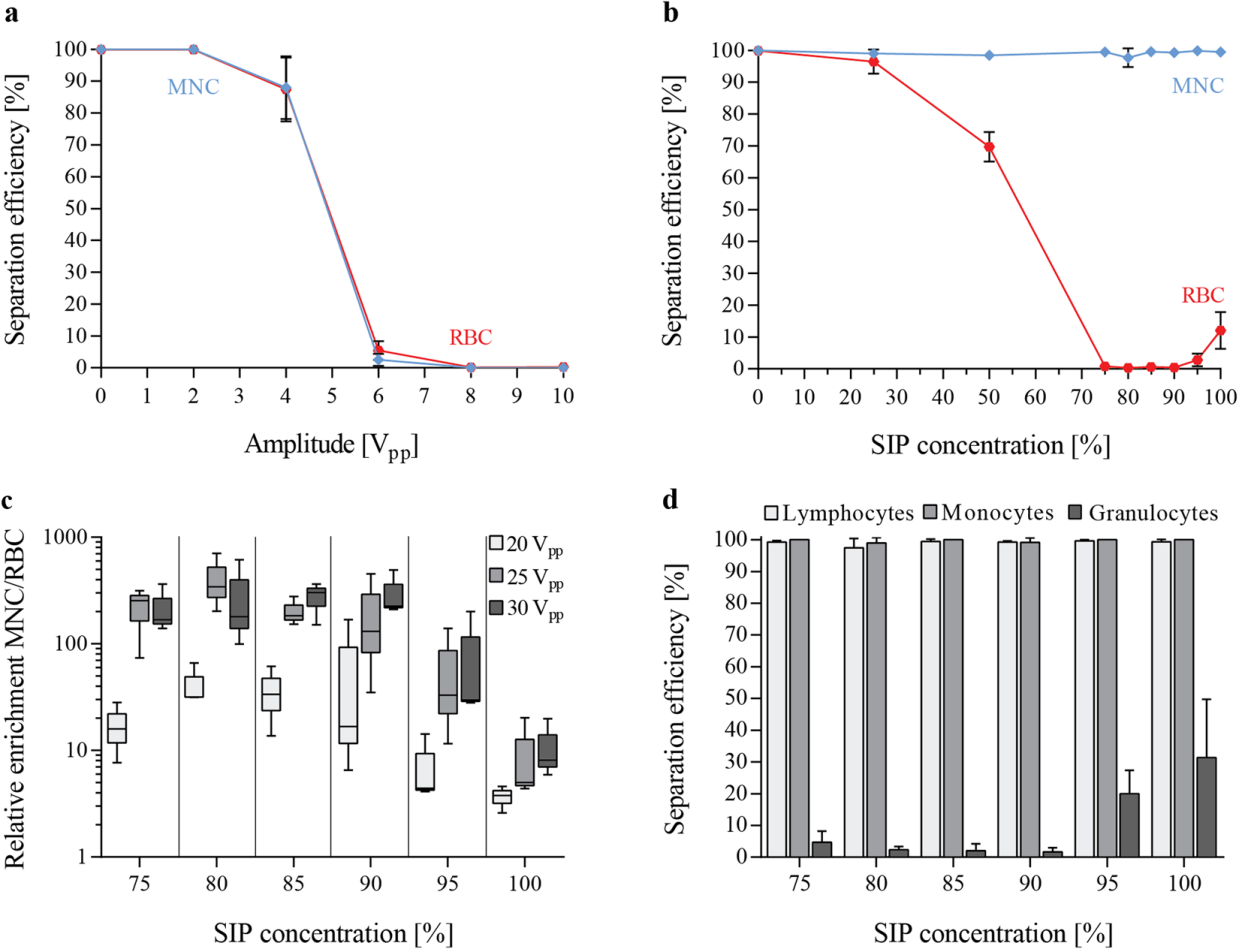
Optimizing buffer condition changes acoustic mobility of blood cells and enables separation of mononuclear cells (MNC) from red blood cells (RBC). (**a**) Separation efficiencies, defined as the ratio of cells in the side outlet as compared to both outlets, are shown for MNCs and RBCs in PBS with increasing strength of the acoustic field, peak-to-peak voltage, V_pp_ (**b**) as well as for varying buffer conditions by increasing the percentage of Stock Isotonic Percoll solution (SIP). A range of 75–100% SIP changes the acoustic mobility of MNCs and RBCs sufficiently to enable separation of the two cell populations. (**c**) The relative enrichment, calculated by the ratio of MNCs to RBCs after the separation compared to the input ratio, MNCs at different SIP concentrations ranging from 75–100% and at different actuation voltages. (**d**) The separation efficiency for different white blood cell populations is shown in the same SIP range for 25 V_pp_. (mean ± SD, n = 3).

The acoustic radiation force acting on a particle in a one-dimensional ultrasonic standing wave field is dependent on particle properties such as size, density and compressibility, and scales with the particle radius to the third power (Supplementary Equation 1). In human whole blood, the cell volumes and densities differ between various populations. Mononuclear cells are larger but less dense (diameter 6–20 µm, volume 160–450 µm^3^, density 1.055–1.070 g/cm^3^) as compared to red blood cells (7–8 µm, 80–100 µm^3^, 1.089–1.100 g/cm^3^)^25,45–47^. Calculations of the acoustophoretic mobility showed an overlap between WBCs and RBCs in PBS (Supplementary Figure 1a), indicating difficulties to acoustophoretically separate WBC or MNC from RBC under these conditions, which was confirmed experimentally. When increasing the magnitude of the acoustic radiation force, both MNC and RBC started to move from the side wall of the acoustophoresis channel (Fig. 1) towards the pressure node in the center fraction, revealing similar separation profiles under standard buffer conditions (Fig. 2a). However, the acoustic radiation force on a cell is also coupled to the acoustophysical properties of the surrounding medium. Changes of the medium properties can thus alter the acoustic forces acting on a cell^35,42,43,48^ as expressed by the acoustic contrast factor^49^. Usually, particles that are denser than the suspending medium display a positive acoustic contrast factor and migrate towards the pressure node in the acoustic field. By increasing the density and decreasing the compressibility of the medium, the acoustic contrast factor of the cell decreases resulting in a reduction of the cell’s acoustophoretic mobility^48^. Ultimately, a movement towards the pressure anti-node can be accomplished if the acoustic contrast factor becomes negative^50^.

By calculating the acoustophoretic mobility for WBC and RBC for modified buffer compositions using increasing concentrations of Stock Isotonic Percoll (SIP) solution, a standard buffer density modifier, we anticipated that the mobility of WBC should approach zero at a concentration of 70% SIP while the corresponding average mobility value for RBC should be about 4000 µm^2^/Pa·s (Supplementary Figure 1a). Based on these calculations, it thus could be expected that MNC would display similar discrimination properties with respect to their acoustophoretic mobility in separation experiments.

We therefore investigated the acoustophoretic mobility of MNC and RBC, as measured by the obtained separation efficiency, in buffer compositions with different SIP concentrations. Dilution of SIP decreases the density of the medium as well as the speed of sound and increases the compressibility (Supplementary Figure 1b,d). For the separation experiments, blood was diluted 100 times in SIP (at indicated concentrations), 500 µl of sample were processed through the acoustic chip, and both outlet fractions were analyzed for their cell content.

For optimum separation, it was critical that the central inlet buffer had the same SIP composition as the buffer used for the cell sample dilution. The acoustic impedance differences between the central buffer and the diluted sample entering through the side inlets, would have otherwise led to an acoustic radiation force-induced translocation of the two fluids, compromising the separation efficiency^51^.

Figure 2b summarizes the outcome of our measurements, showing the separation efficiency for MNC and RBC at different SIP concentrations. The amplitude settings on the 2 MHz transducer were adjusted to the different acoustophoretic conditions as follows: 0% SIP (equivalent to PBS) 2V_pp_, 25% SIP 4V_pp_, 50% SIP 6V_pp_, 75–85% SIP 25V_pp_, and 90–100% SIP 30V_pp_. Based on the theoretical calculations (Supplemental Figure 1A) both MNCs and RBCs displayed a lower acoustophoretic mobility at increased SIP concentrations. Therefore, voltage settings had to be increased in order to achieve suitable pressure amplitudes, to move the RBCs equivalently into the center fraction at higher SIP concentrations. Scanning of the optimal voltage range was done for the different buffer conditions (Supplementary Figure 2). For 0–25% SIP, no separation was possible regardless of the voltage, which is due to the similar acoustophoretic mobility of MNCs and RBCs under these conditions (Supplementary Figure 2). At a concentration of 50% SIP, the acoustophoretic mobility of the MNCs was reduced in relation to RBCs, resulting in a mean separation efficiency (±SD) of 98.4 ± 1.2% of MNCs while 30.3 ± 4.7% of the RBCs were depleted. Optimal separation was achieved between 75–100% SIP, with RBC contamination rates of only 0.8 ± 0.6% and MNC separation efficiencies of 99.4 ± 0.4% at 75% SIP. Increasing the SIP concentration to 100% also reduced the mobility of RBCs which was reflected by a decreased RBC depletion rate to 88.0 ± 6.7% (Fig. 2b).

For SIP concentrations above 90%, RBCs did not have sufficient time to migrate into the center fraction before reaching the outlets, resulting in a decreased RBC depletion rate. Theoretically, this decrease in RBC depletion could be compensated by decreasing the flow rate, which however would also decrease the throughput. Alternatively, increasing the length of the main separation channel would enable the RBCs to reach the center fraction even at SIP concentrations >90%. Accordingly, design-modified chips, as well as increased amplitude of the acoustic field could further decrease the RBC contamination when using high SIP concentrations. However, increasing the amplitude significantly increases temperature losses in the piezoelectric actuator, causing a temperature drift of the system, and ultimately leading to a drift in the resonance frequency of the acoustophoresis channel^52^.

The acoustic separation outcome can be expressed as the relative enrichment of MNC to RBC, calculated by MNC to RBC cell ratio after the separation divided by the corresponding input ratio. Figure 2c illustrates the results for SIP concentrations between 75–100% for three different actuation voltages. The highest enrichment was achieved at 80% SIP, 25V_pp_ with a mean (±SD) of 390.6 ± 169.8-fold enrichment. Under these conditions, increasing the voltage to 30V_pp_ resulted in a higher fraction of MNCs that was translocated to the center, thus resulting in a decreased relative enrichment. In comparison, for higher SIP concentrations, more RBCs contaminated the side outlet fraction leading to a lower relative enrichment.

Lymphocytes and monocytes, as part of the MNCs, together with granulocytes are subpopulations of white blood cells. Both lymphocytes and monocytes show a similar behavior in the acoustic field and can be collected in the side outlet with high separation efficiencies at SIP concentrations ranging between 75–100% SIP and 25V_pp_ actuation (Fig. 2d). In contrast, granulocytes display acoustophysical properties similar to RBCs and are therefore moved towards the acoustic pressure node in the microchannel center. At SIP concentration of 75–90% less than 5% of all granulocytes are collected in the side outlet fraction, resulting in a less contaminated and more purified MNC sample.

### Blood concentration influences separation efficiency

**Figure 3:**
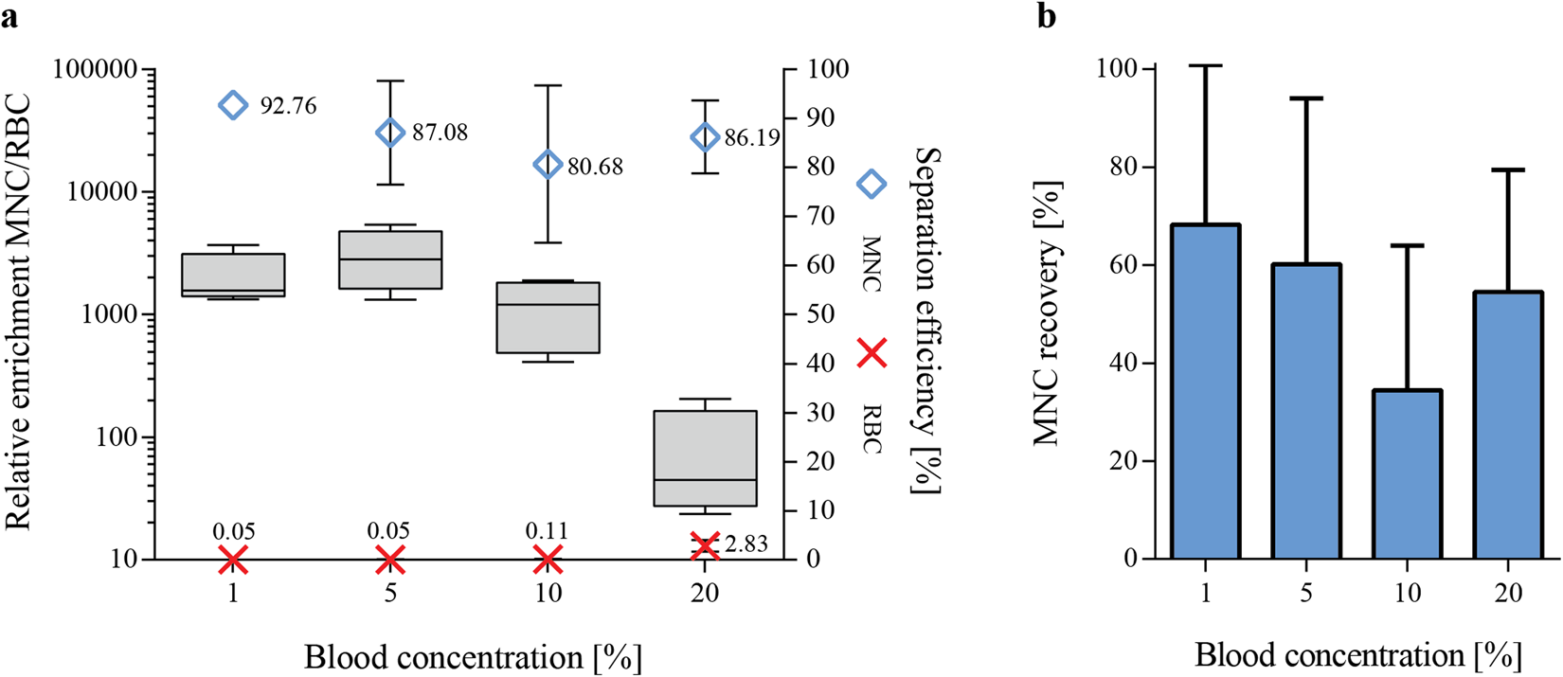
Whole blood dilution rates influenced separation outcome. (**a**) The separation efficiencies of MNC (blue) and RBC (red) in the side outlet fraction as well as the relative enrichment of MNC to RBC as compared to the input blood sample (boxplot) for different blood concentrations are shown (n = 4). (**b**) The recovery of MNC for the different blood concentrations was calculated as the percentage of cells in the output to the input sample. Blood was diluted in 80% SIP and separated at 25 V_pp_ (mean ± SD, n = 4).

When using external force fields in microfluidic separations, the particle concentration becomes a critical factor for the separation outcome, as also confirmed by theoretical modeling of hydrodynamic particle-particle interactions^53,54^. At high particle concentrations, the hydrodynamic interaction between particles increases, which causes the suspension to move as a whole. For a given particle, the migration velocity towards the pressure node is lowered at higher particle concentrations at the same time as other particles with lower acoustophoretic mobility are hydrodynamical coupled to faster moving particles. The threshold for hydrodynamic coupling, estimated by monitoring the washing efficiency of beads or cells, has experimentally been shown to range to between 10^7^–10^8^ particles/mL^55–57^ depending on the particle size used. The theoretical estimates by Ley *et al*.^54^ on the threshold values for hydrodynamic coupling in acoustophoresis systems indicate that volume fractions above 0.01 significantly impact the separation. Human undiluted blood has an average of about 5 × 10^9^ cells/ml (volume fraction ≈0,4) which is clearly above the threshold for hydrodynamic coupling. Therefore, the influence of different starting blood concentrations diluted in 80% SIP on the acoustophoretic separation outcome was investigated. A 200 µl sample was separated at 25V_pp_ and the separation efficiencies of MNCs and RBCs for the side outlet fraction, as well as the relative enrichment of MNCs to RBCs as compared to the input sample were recorded. Dilution of blood decreased the average cell concentration to about 5 × 10^8^ cells/ml (10% blood, volume fraction ≈0,04) and 5 × 10^7^ cells/ml (1% blood, volume fraction ≈0,004). As shown in Fig. 3a, an increase in the final blood concentration led to a decrease in mean separation efficiency (±SD) of MNC from 92.8 ± 1.3% at a blood concentration of 1% to 80.7 ± 16.1% at 10% blood concentration, in agreement with the theoretical threshold for hydrodynamic coupling. Interestingly, increasing the starting blood concentration further to 20% resulted in a slightly higher mean MNC separation efficiency (86.2 ± 7.4%) compared to 10%. However, in this case also more RBCs were recovered in the side outlet due to an overload of the central outlet, an effect which has been reported previously for washing of blood cells^33^. Overall, the best results with a median relative enrichment of MNC to RBCs of 2806-fold (range 1318–5398) were achieved for a starting blood concentration of 5% (Fig. 3a).

Acoustophoretic separation of blood cells outperforms other previously reported continuous and label-free microfluidic separation devices, both in relative enrichment as well as sample throughput. The published literature reports sample flow rates between 0.018 µl/min and 3.6 µl/min undiluted whole blood equivalent, however, relative enrichment rates of WBCs versus RBCs are often as low as 15–110 fold^8,9,13,16,21,23^ compared to 2,800-fold of MNC enrichment with our system. The most promising enrichment results obtained with other systems were reported for a microfluidic cross-flow device^8^, which enabled to increase the ratio of WBC to RBC 2,000-fold as compared to the initial sample with ~98% separation efficiency while recovering ~52% of the WBC. However, the overall throughput of this system reached only 0.06 µl/min of undiluted whole blood equivalent, which is more than 80 times lower compared to the acoustophoretic system reported herein. Unfortunately, many studies in the field only report separation results for the whole WBC population^8,13,16,21,22^ without considering that different WBC subtypes might respond differently to the separation process and thus introduce a possible bias. This makes a direct comparison of the published methods to acoustophoretic MNC separation difficult. Nevertheless, in our study, a sample throughput of 5 µl/min whole blood equivalent (>10^5^ cells/s) was achieved when using a 5% blood concentration.

When calculating the overall enrichment of WBC (accounting for the depletion of granulocytes of 82.1 ± 11.5%) a median 1,013-fold enrichment of WBCs to RBCs was accomplished, which is considerably higher than most other reported microfluidic systems.

The total MNC recovery, as calculated by the percentage of mononuclear cells in the side outlet compared to the input cell amount, was lower than the measured separation efficiency (Fig. 3b). However, this can be explained by the residual volume of the microfluidic system, such as connecting tubings and the microfluidic chip, in which a significant fraction of the sample volume remains after processing. Obviously, this problem becomes less relevant when higher volumes are processed and can easily be solved by introducing a final flushing step. This is illustrated by running 200 µl sample of 1% blood which produced a total MNC recovery of 68% whereas MNC recovery was increased to 88% by increasing the volume to 500 µl, which resulted in separation efficiency of 92%.

### Acoustic separation in comparison to standard separation methods

**Figure 4:**
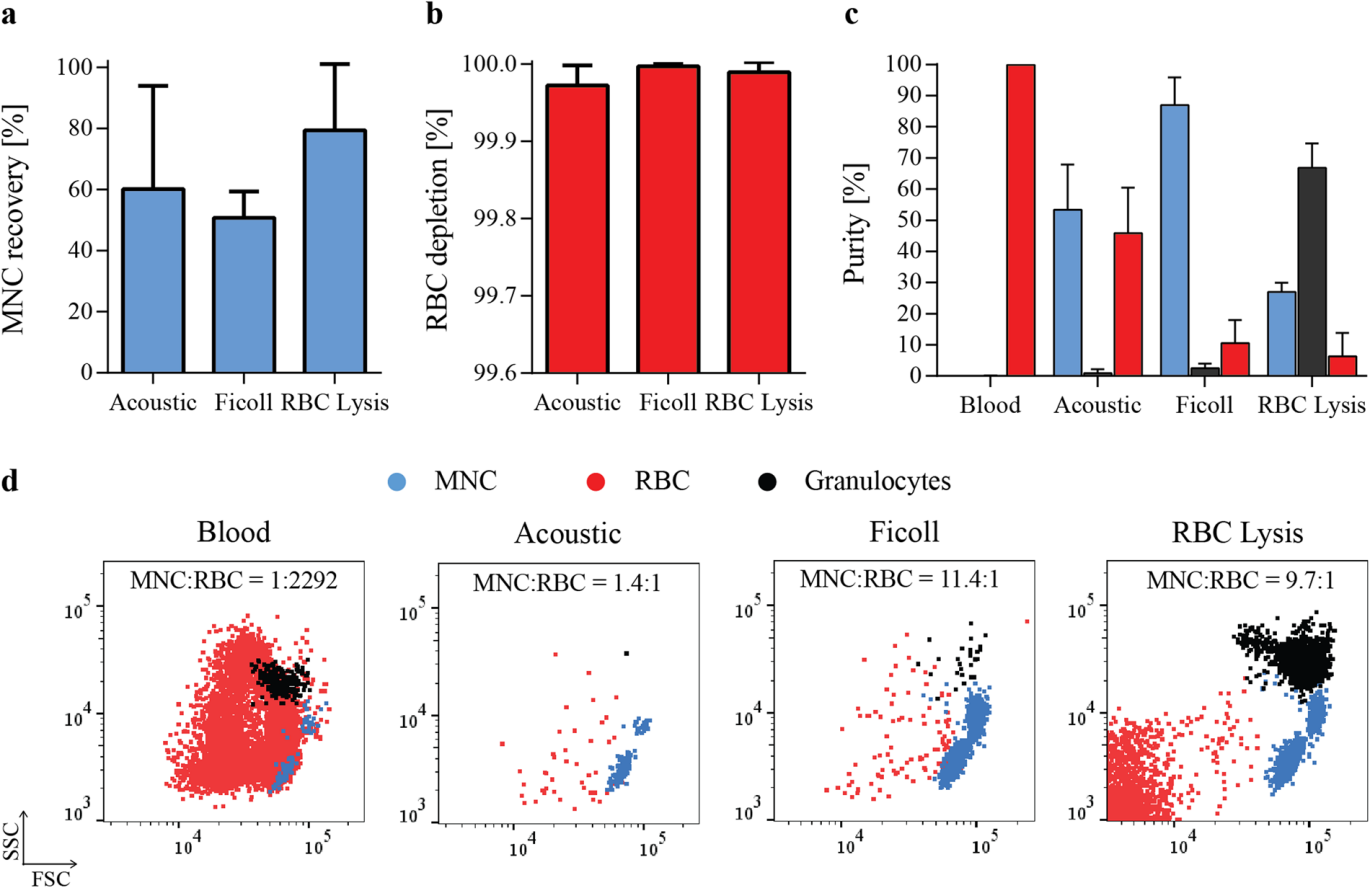
Comparison of acoustophoresis and standard separation methods. (**a**) Recovery of MNCs, calculated as the percentage of cells in the output compared to the input cell amount, and (**b**) depletion of RBCs are shown for acoustic separation as well as for density gradient centrifugation (Ficoll) and RBC lysis (mean ± SD, n = 3). (**c**) Purities of the different populations as obtained by each separation method were calculated as the ratio of MNCs (blue), granulocytes (black) and RBCs (red) respectively to the total number of cells in the separated sample (mean ± SD, n = 3). (**d**) Representative FACS plots show the distribution of the different cell types in the starting blood sample (left) as well as after the different separation methods.

Separation of MNCs can be performed by various methods, all of them having their benefits and disadvantages. Here, we compared the acoustophoretic separation to two standard methods, Ficoll separation and RBC Lysis. Isolation of MNCs using Ficoll is based on differences in cell density using density gradient centrifugation, whereas RBC Lysis selectively disrupts RBCs in the blood sample followed by washing and enrichment of MNCs by sequential centrifugation steps. MNC recovery and RBC depletion are shown for the different separation methods in Fig. 4a and b, respectively. All three methods reached comparable RBC depletion rates of over 99.97%. The lowest mean (±SD) recovery of MNCs of 50.7 ± 8.7% was achieved using Ficoll separation, which also required the largest volume of blood (5 mL). The highest recovery was obtained using RBC lysis with 79.4 ± 21.7%. As described previously, MNC recovery for acoustic separation is dependent on the starting blood concentration as well as on the processed sample volume and was highest for 500 µl of 1% starting blood concentration (88.5 ± 15.6%). Figure 4 shows the results for acoustic separation of blood at 5% starting concentration, 200 µl sample volume, run with 80% SIP at 25V_pp_ which gave the highest relative enrichment of MNCs to RBCs (Fig. 3a).

Sample purities (Fig. 4c) as well as representative FACS plots (Fig. 4d) show the different distributions of cell types in the input and output samples. Starting blood samples consisted to 99.86 ± 0.03% of RBCs, to 0.1 ± 0.03% of granulocytes and only to 0.044 ± 0.005% of MNCs, which corresponded to a MNC to RBC ratio of 1 to 2292. Acoustic separation considerably reduced the number of RBCs leading to a MNC to RBC ratio of 1.4 to 1, with a MNC purity of 53.3 ± 14.5%. In comparison, Ficoll density gradient centrifugation resulted in the highest ratio in MNC to RBCs of 11.5 to 1 and a MNC purity of 87 ± 8.9%. RBC lysis had the highest granulocyte contamination and gave the lowest purity of MNCs of 27 ± 3% but also the lowest RBC contamination with a MNC to RBC ratio of 9.7 to 1. Furthermore, RBC lysis increased the amount of cell debris in the sample as indicated in the flow cytometry data (Fig. 4d).

Based on our results, acoustophoresis offers an alternative to standard MNC separation methods, and can also be applied to separate other cell types which show overlapping acoustic mobilities in standard buffer conditions. Furthermore, due to the continuous acoustic separation of MNC from blood, the device can be easily integrated with downstream applications, such as direct analysis of MNC in a flow cytometer. Counting MNC from whole blood with cell concentrations of ~5.000.000 cells/µl is not possible with current flow cytometer systems (flow speed of 10.000–200.000 events/s, cell concentrations 5.000–1.000.000 cells/µl)^58^. Acoustophoresis can decomplex blood samples sufficiently from ~115.000 cells/s to ~560 cells/s and thus enable analysis of a purified MNC fraction directly in the flow cytometer without increasing the number of manual steps such as pipetting or centrifugation.

## Conclusion

We have shown that acoustophoresis can be used to enrich mononuclear cells from red blood cells with high efficiency and recovery. In agreement with theoretical calculations we demonstrate experimentally that the acoustophoretic mobilities of MNC and RBC can be differentially affected by modifying the acoustophysical properties of the buffer, thereby enabling efficient separation of the two cell types with otherwise overlapping acoustophoretic mobilities.

## Materials and Methods

### Ethical statement

The collection of blood samples from healthy volunteers was approved by the Regional Ethical Review Board at Lund University. All experiments were performed in accordance with relevant guidelines and regulations.

### Sample collection

Blood samples were obtained from healthy volunteers after informed consent at Lund University Hospital, Lund, Sweden and collected in vacutainer tubes (BD Bioscience, San Jose, CA, USA), containing ethylenediaminetetraacetic acid (EDTA) as anticoagulant.

### Central inlet buffer preparation

Percoll density medium (Sigma-Aldrich, St. Louis, MI, USA) was used as central inlet buffer as well as to prepare dilutions of blood for the acoustophoretic separation. To adjust the osmolality of undiluted Percoll, 1 part (v/v) of 1.5 M NaCl (Sigma-Aldrich) was added to 9 parts (v/v) of Percoll to make a stock isotonic percoll (SIP) solution. Adjustments of the density of SIP were done by further dilution of SIP in 0.15 M NaCl.

### Sample preparation, immunostaining and flow cytometric analysis

Blood samples were processed within 10 hours after collection, stained with monoclonal antibodies for 15 min at room temperature in the dark and further diluted in central inlet buffer. The following directly conjugated monoclonal antibodies were used in this study: CD3-APC (clone HIT3a), CD14-PE (clone MφP9), CD19 FITC (clone HIB19), CD45-PerCP (clone 2D1), CD61-PE (clone VI-PL2), CD66b FITC (clone G10F5) and CD235a APC (clone GA-R2), as well as matched isotype controls (all from BD Bioscience, San Jose, CA, USA). Stained samples were analyzed before and after separation on a FACSCanto II flow cytometer (BD Bioscience) and acquired data was further analyzed using the FlowJo software (Tree Star Inc., Ashland, OR, USA).

### Standard methods for mononuclear cell separation

Mononuclear cells (MNC) were isolated by Ficoll density gradient centrifugation using Ficoll-Paque Premium (1.078 g/ml, GE Healthcare Life Sciences, Little Chalfont, UK). In brief, 5 mL blood was diluted four times and layered over 15 mL of Ficoll-Paque Premium. After 40 min of centrifugation at 400 × g the mononuclear cell layer was removed and washed twice at 200 × g, for 10 min.

For comparison, selective lysis of red blood cells (RBCs) to enrich the MNC fractions was performed using BD Pharm Lyse (BD Bioscience, San Jose, CA, US). A total of 200 µL blood was lysed by adding 2 mL of 1x lysing solution, incubated for 15 min at room temperature in the dark and washed twice at 200 × g for 5 minutes.

### Acoustophoretic setup

#### Microfluidic chip design

The acoustophoretic chip was manufactured by Micronit (Enschede, Netherlands) using Deep Reactive Ion Etching and sealed with a glass lid. The channel structure was etched into silicon and consists of a sample inlet, a pre-focusing zone (26 mm × 300 µm × 150 µm), a v-shaped flow splitter around the central buffer inlet, a main separation channel (43 mm × 375 µm × 150 µm) and a trifurcation with a central outlet and a common side outlet for the two lateral branches (Fig. 1).

#### Acoustic actuation

The standing wave field was created using piezoceramic transducers glued underneath the pre-focusing channel as well as underneath the main separation channel. Frequencies were set to 4.831 MHz for the pre-focusing channel with 5 V_pp_ amplitude and 1.956 MHz for the main separation channel. A dual channel function generator (AFG3022B, Tektronix, Beaverton, OR, USA), connected to signal amplifiers (in-house build) was used to drive both transducers while the voltage over each transducer was measured via a two-channel digital oscilloscope (TDS 1002, Tektronix). Temperature regulation was achieved using a Peltier element and a PT100 resistance temperature detector attached to the acoustophoretic system.

#### Fluidic setup and sample procedur

An in-house designed pressure driven system with feedback regulation was used for controlling the fluids to and from the chip monitored by SLI-1000 Liquid Flow Meters (Sensirion AG, Switzerland)^59^. A total volume of 200–500 µl diluted blood was infused through the sample inlet at 100 µl/min and pre-aligned in width and height into two parallel bands in the pre-focusing channel. Pre-aligned blood entered the main-separation channel, with a central inlet buffer flow rate set to 300 µl/min, and cells were separated in the acoustic standing wave field, based on their acoustophysical properties into the center outlet fraction (100 µl/min) or the side outlet fraction (300 µl/min). Samples of both outlet fractions were collected in 5 ml tubes and analyzed for their cell content.

### Data availability

The authors declare that all data supporting the findings of this study are provided in the paper and it’s Supplementary Information. Raw data is available on request.

## Electronic supplementary material

Supplementary Information
