# Supplementary material for: Rapid and effective enrichment of mononuclear cells from blood using acoustophoresis
Source: Sci Rep. 2017 Dec 7;7:17161. doi: 10.1038/s41598-017-17200-9 (PMC5719459; doi:10.1038/s41598-017-17200-9)
Supplement: Supplementary file 1 — Supplementary Information [file 41598_2017_17200_MOESM1_ESM.pdf]

Rapid and effective enrichment of mononuclear cells from blood using acoustophoresis

Anke Urbansky[1,*], Pelle Ohlsson[1,2], Andreas Lenshof[1], Fabio Garofalo[1], Stefan Scheding[3,4,5] and Thomas Laurell[1,6]

[1]Department of Biomedical Engineering, Lund University, 221 00 Lund, Sweden. [2]AcouSort AB, BMC D10, 221 84 Lund, Sweden. [3]Lund Stem Cell Center, Lund University, 221 00 Lund, Sweden. [4]Division of Molecular Hematology, Department of Laboratory Medicine, Lund University, 221 00 Lund, Sweden [5]Department of Hematology, Skåne University Hospital, 222 41 Lund, Sweden. [6]Department of Biomedical Engineering, Dongguk University, 04620 Seoul, Korea. *e-mail: anke.urbansky@bme.lth.se

Supplementary

$$F_z^{rad} = \frac{4}{3}\pi\phi(\tilde{\kappa},\tilde{\rho})ka^3 E_{ac}\sin(2kz)$$

$$E_{ac} = \frac{p_a^2}{4\rho_0 c_0^2}\,; \quad \phi(\tilde{\kappa},\tilde{\rho}) = \frac{5\tilde{\rho}-2}{2\tilde{\rho}+1} - \tilde{\kappa}; \qquad \tilde{\kappa} = \frac{\kappa_p}{\kappa_0}; \qquad \tilde{\rho} = \frac{\rho_p}{\rho_0}$$

Supplementary Equation 1. Acoustic radiation force F_z^{rad} acting on a particle in an acoustic standing wave field where κ_0, ρ_0, κ_p and ρ_p are the compressibility and density of the fluid and particle, $\phi(\tilde{\kappa},\tilde{\rho})$ is the acoustic contrast factor, k is the wave number ($2\pi/\lambda$), E_{ac} is the acoustic energy density, z is the position of the particle along the wave propagation axis, p_a is the pressure amplitude, c_0 is the speed of sound in the medium[1].

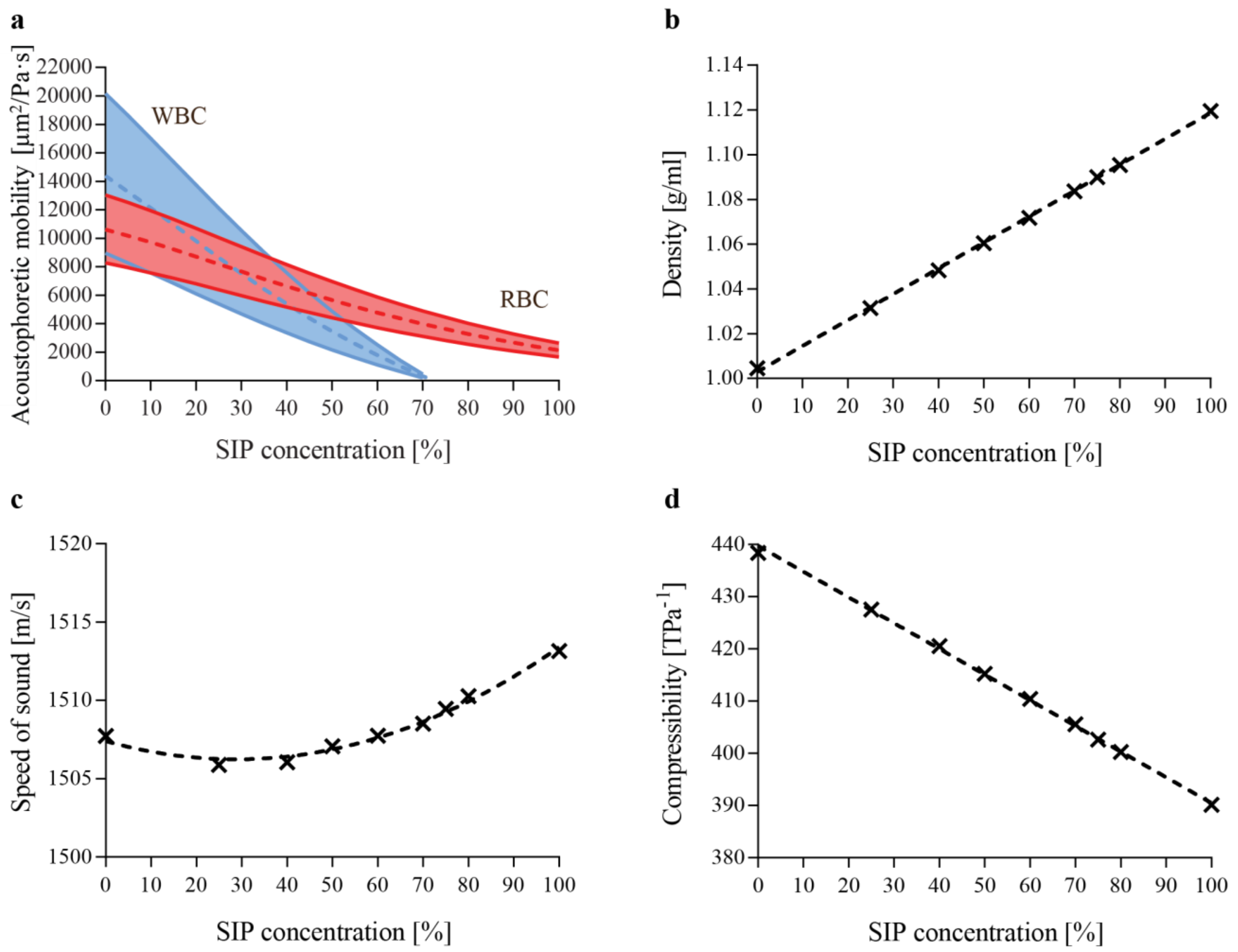

Supplementary Figure 1. (**a**) Acoustophoretic mobility, calculated as the radius squared times the acoustic contrast factor divided by the viscosity of the medium, $a^2\Phi/\eta$, is shown for WBC (blue) and RBC (red) at different concentrations of stock isotonic percoll solution (SIP) with the colored area showing the variance associated with the size variability of the population and the dashed line indicating the average mobility value (calculations are based on values obtained by Cushing et al.[2]). (**b**) Both the density ρ and (**c**) speed of sound c at 25°C of SIP (black) as a function of the SIP concentration were determined using a density and sound velocity meter (DSA 5000M, Anton Paar GmbH). (**d**) The compressibility κ was calculated from the obtained values as $1/(\rho c^2)$.

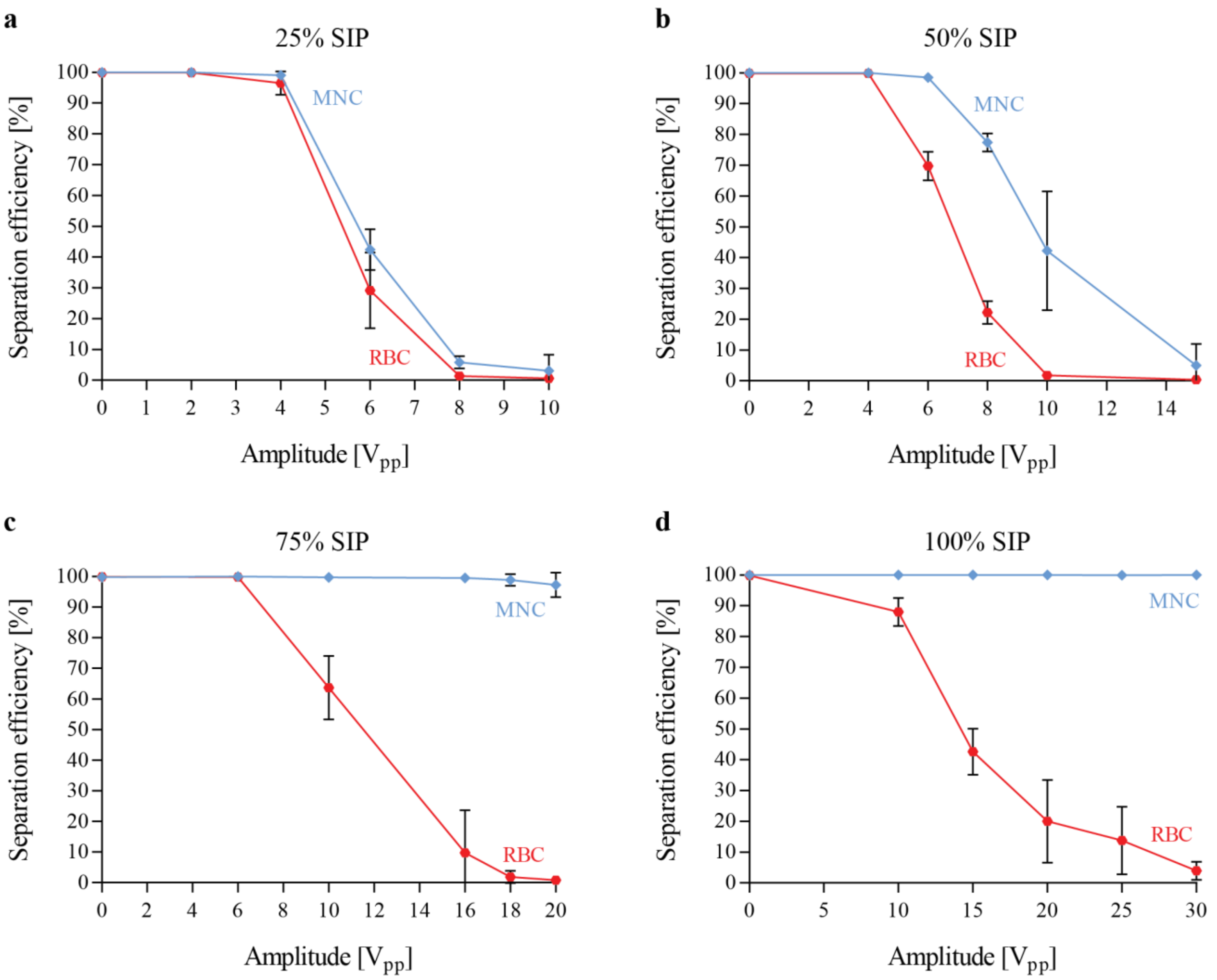

Supplementary Figure 2. Increasing concentrations of SIP enabled the acoustic separation of MNC and RBC. Blood was diluted in increasing concentrations of stock isotonic percoll solution (SIP) of (**a**) 25%, (**b**) 50%, (**c**) 75%, and (**d**) 100%, and perfused through the acoustophoretic chip at varying amplitudes of the acoustic field. Separation efficiency, defined as the ratio of cells in the side outlet as compared to both outlets, is shown for mononuclear cells (MNC) and red blood cells (RBC). (n=3)

References

1. Laurell, T. & Lenshof, A. *Microscale Acoustofluidics*. (Royal Society of Chemistry, 2014).
2. Cushing, K. W. *et al.* Ultrasound Characterization of Microbead and Cell Suspensions by Speed of Sound Measurements of Neutrally Buoyant Samples. *Anal. Chem.* **89,** 8917–8923 (2017).
